# Reusable Standardized Universal Interface Module (RSUIM) for Generic Organ-on-a-Chip Applications

**DOI:** 10.3390/mi10120849

**Published:** 2019-12-05

**Authors:** Qiyue Sun, Jianghua Pei, Qinyu Li, Kai Niu, Xiaolin Wang

**Affiliations:** 1 Department of Micro/Nano Electronics, School of Electronic Information and Electrical Engineering, Shanghai Jiao Tong University, Shanghai 200240, China; kizluy@sjtu.edu.cn (Q.S.); Peijianghua@sjtu.edu.cn (J.P.); cosmos22@sjtu.edu.cn (Q.L.); nk_sjtu@sjtu.edu.cn (K.N.); 2 National Key Laboratory of Science and Technology on Micro/Nano Fabrication, Department of Micro/Nano Electronics, School of Electronic Information and Electrical Engineering, Shanghai Jiao Tong University, Shanghai 200240, China; 3 Key Laboratory of Thin Film and Microfabrication Technology (Ministry of Education), Department of Micro/Nano Electronics, School of Electronic Information and Electrical Engineering, Shanghai Jiao Tong University, Shanghai 200240, China; 4 Institute of Medical Robotics, Shanghai Jiao Tong University, Shanghai 200240, China

**Keywords:** microfluidics, vascularization, organ-on-a-chip, tissue engineering, interface module

## Abstract

The modular-based multi-organ-on-a-chip enables more stable and flexible configuration to better mimic the complex biological phenomena for versatile biomedical applications. However, the existing magnetic-based interconnection modes are mainly realized by directly embedding and/or fixing magnets into the modular microfluidic devices for single use only, which will inevitably increase the complexity and cost during the manufacturing process. Here, we present a novel design of a reusable standardized universal interface module (RSUIM), which is highly suitable for generic organ-on-chip applications and their integration into multi-organ systems. Both pasting-based and clamping-based interconnection modes are developed in a plug-and-play manner without fluidic leakage. Furthermore, due to the flexibility of the modular design, it is simple to integrate multiple assembled modular devices through parallel configuration into a high throughput platform. To test its effectiveness, experiments on the construction of both the microvascular network and vascularized tumor model are performed by using the integration of the generic vascularized organ-on-a-chip module and pasting-based RSUIM, and their quantitative analysis results on the reproducibility and anti-cancer drug screening validation are further performed. We believe that this RSUIM design will become a standard and critical accessory for a broad range of organ-on-a-chip applications and is easy for commercialization with low cost.

## 1. Introduction

Human organ-on-a-chip systems have become useful and powerful tools for the integration of human cells/microtissues and hydrogels into microfluidic devices with multiple microphysiological environment controls to recapitulate the vivo-like 3D microstructures and specific-organ functions [1]. As an alternative to the conventional cell culture with 2D monolayer or animal models with species differences, organ-on-a-chip technologies show enormous potentials in the fields of drug development, personalized medicine, and disease modeling [2,3].

Generally, organ-on-a-chip systems can be categorized into two different concepts. The first is the single organ-on-a-chip with one type of specific tissue, such as lung-on-a-chip [4], gut-on-a-chip [5], liver-on-a-chip [6], heart-on-a-chip [7]. It can be further divided into generic platforms for various types of tissues through a one-fits-all solution [4,5], as well as the specific-organ platforms with only one tissue type [7]. The second is the multi organ-on-a-chip by integrating multiple organs into one platform to represent the blood circulation and tissue-to-tissue communications, which can be further categorized into static, semi-static, and modular designs. For the static design, the geometry of tissue chambers is fixed into a predefined order, which needs the simultaneous extracellular matrix (ECM) loading at the beginning of experiment and culturing with the common universal medium [8]. The semi-static designs are often based on the tissue interconnection through the integrated microfluidic channels with Transwell^®^-based tissue inserts [9]. For the modular design, individual specific/generic single organ-on-a-chip can be integrated through interconnectors or tubings with high flexibility after their maturation at any given time, which enables the ECM loading and tissue culture separately and individually with the tissue-specific culture medium. Therefore, due to high temporal flexibility and redundancy capacity, the modular-based multi organ-on-a-chip design can lead to a more stable and viable configuration based on specific applications compared with both the static and semi-static designs [10].

Although there are enormous potentials for the flexible modular-based multi organ-on-a-chip design, significant engineering challenges still exist, especially in the standardization of multi-organ systems. This involves standardizing the individual modular organ-on-a-chip and the world-to-chip interface, as well as the interorgan connectors. The configurable plug-and-play concept-based modular microfluidic chip is commonly used for building multi organ-on-a-chip systems, which can be easily assembled, disassembled, and reconfigured again. In all these configurations, the critical issue is how to realize the modular-to-modular microfluidic interconnection without leakage. These interconnects or interfaces should be simple to use, easy to manufacture, reversible, and, most importantly, reliable after repeated assembly and disassembly. Earlier LEGO^®^ concept-based microfluidic modular designs were designed based on either LEGO bricks by micromilling small channels [11] or polydimethylsiloxane (PDMS)-based building blocks casted from 3D-printed master molds [12] and then integrated into microfluidic systems using the popular interlocking blocks. However, through this design, it will be difficult to fabricate the modular microfluidic chip with complex microfluidic channels as well as tissue culture chambers for organ-on-a-chip applications. Besides the LEGO-like design, the magnets were also utilized as the universal interlocking connectors for reconfigurable modular microfluidic systems. Yuen proposed the magnetic interconnects by comprising ring magnets and sealing gaskets at each module inlet and outlet that was positioned at the horizontal surface [13]. In order to interconnect them, one modular device had to be flipped so that it would be impossible for imaging at the same plane. To circumvent this limitation, Ong et al. developed a self-aligning Tetris-like (TILE) modular microfluidic platform by designing the magnetic interconnects at the lateral surfaces of microfluidic device [14]. Although the equiplanar connection between separate TILE modules was realized for easy imaging, the magnets were embedded into the PDMS-based modules for single use only, which would increase the complexity and cost during the manufacturing process.

In this paper, we have developed a reusable standardized universal interface module (RSUIM) with high flexibility, which is highly suitable for generic organ-on-a-chip applications and their integration into multi organ-on-a-chip systems. Different from previous interconnects directly embedded and/or fixed on different microfluidic chip modules, the RSUIM is an independent unit, constructed by incorporating both ring magnet and sealing gasket into a poly(methyl methacrylate) (PMMA)-based module, which could be utilized multiple times. Furthermore, both pasting-based and clamping-based RSUIM is developed to establish the interconnection between the RSUIM and organ-on-a-chip module without fluidic leakage. In addition, due to the modular design, the assembled RSUIM with versatile organ-on-a-chip modules can be interconnected with other functional modules through either serial or parallel configurations with high flexibility. To test the effectiveness of this RSUIM, the experiments on vascularized microtissue formation and anti-cancer drug validation were further performed and quantitively analyzed.

## 2. Materials and Methods

### 2.1. Pasting-based RSUIM Design

**Figure 1:**
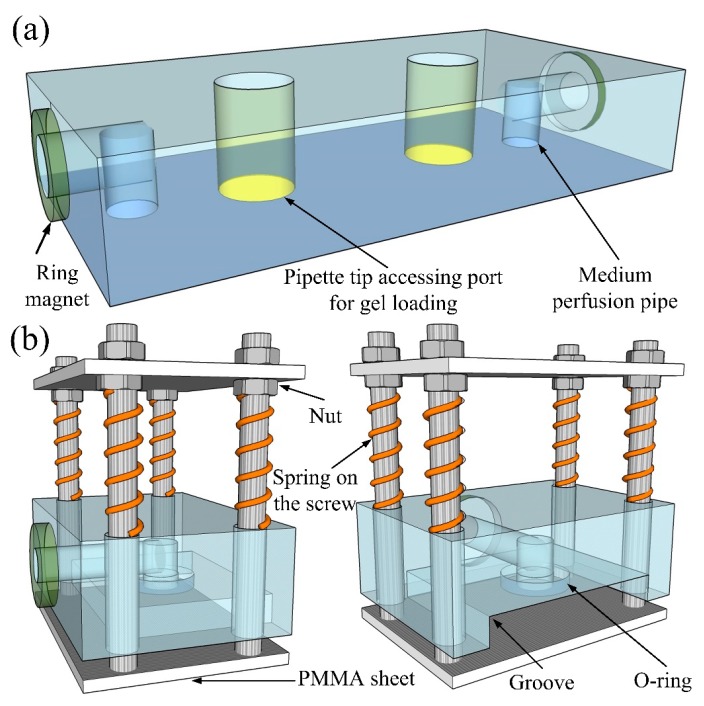
Schematic of two different types of reusable standardized universal interface module (RSUIM) design. (**a**) Pasting-based RSUIM design, (**b**) clamping-based RSUIM design.

Figure 1a shows the schematic of the whole pasting-based RSUIM design. Briefly, two nickel-plated neodymium ring magnets (N52 8 mm outside diameter (OD) × 3 mm inside diameter (ID) × 1.5 mm thick, Magnet Tiger, Ningbo, China) with opposite polarities (either the north or the south pole) were individually press-fitted on the side of the RSUIM and glued into each recess with epoxy for the tight adhesion to prevent detaching during the separation process. Here, the thickness of the ring magnet (1.5 mm) was slightly larger than the depth of the recess (1 mm), leading to a small protrusion of magnet to be extended out of the recess to ensure the feasibility of complete interconnection. The horizontal medium perfusion pipe with a diameter of 3 mm was then drilled from the center of the ring magnet on both sides of the RSUIM and the vertical medium perfusion pipe with the same diameter was drilled at the corresponding position of the medium inlets and outlets on organ chips, which ultimately formed the 90° elbow medium access ports. Finally, holes at the pipette tip accessing the port with a large diameter, such as 4 mm, were dilled at the position of the gel loading inlet and outlet on organ chips for easy ECM loading.

### 2.2. Clamping-based RSUIM Design

The schematic of the clamping-based RSUIM design is shown in Figure 1b. Both the magnetic interconnection and medium perfusion pipe were the same as that in the pasting-based RSUIM design. In addition, four holes were drilled at the corners of the PMMA module to facilitate the fixing with four screws and two PMMA sheets. The upper PMMA sheet was fixed between two nuts, and the lower sheet was screwed and bonded with epoxy. Between the upper surface of this PMMA module and the nut below the upper PMMA sheet, four springs with an inner diameter slightly larger than the screw were placed, which could flexibly move the lower PMMA sheet up and down to complete the clamping of the organ-on-a-chip module. Furthermore, in order to realize the robust fixing and tight interconnection, a groove with an O-ring (8 mm OD × 5 mm ID × 1.5 mm thick) was fabricated below the PMMA module, whose depth was smaller than that of the organ-on-a-chip module. In addition, the thickness of the O-ring was larger than the depth of the recess (1 mm) on the groove, which could prevent fluidic leakage during medium perfusion.

### 2.3. Generic Vascularized Organ-on-a-Chip and Medium Reservoir Modules

**Figure 2:**
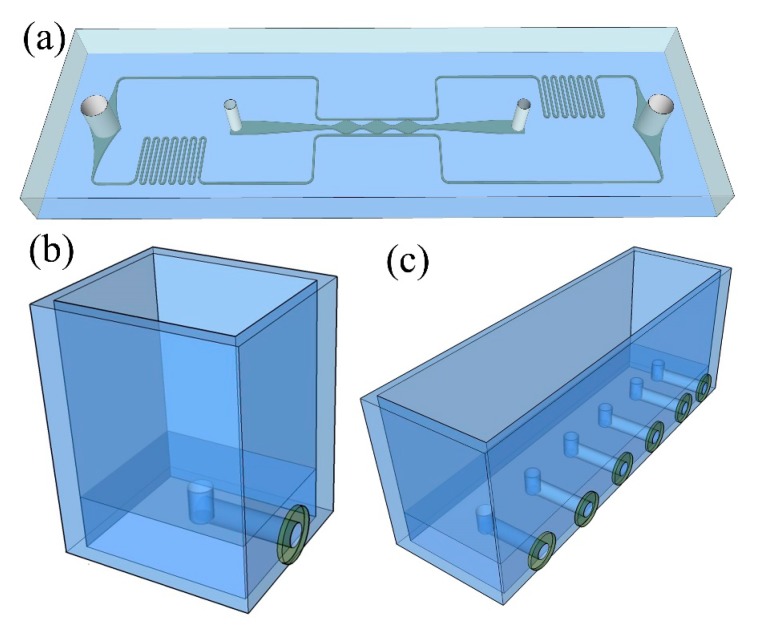
Schematic of the generic vascularized organ-on-a-chip and medium reservoir modules design. (**a**) Coupling design of the microfluidic chip with two medium perfusion ports and two gel loading ports. (**b**) Medium reservoir module with only one medium access port. (**c**) Medium reservoir module with six medium access ports.

The design of the generic vascularized organ-on-a-chip was adopted from previous studies [15,16], as shown in Figure 2a. Briefly, the dimensions of this modular chip were fixed at 5 cm length, 2 cm width, and 3 mm height, which directly determines the parameters of the RSUIM design. The entire microfluidic chip structure consisted of three central millimeter-sized diamond tissue chambers (1 × 2 mm) connected to two-sided square cross-sectional microfluidic channels (100 × 100 μm) through 50 μm wide capillary burst valves. Two ports for medium perfusion and two ports for gel loading were positioned at the end of the microfluidic channel and tissue culture chamber, respectively. The coupling design with the asymmetrical microfluidic channel could generate the interstitial flow to stimulate the ECM inside the tissue chambers. By co-culturing different microtissues with the microvascular network, versatile vascularized organ-on-a-chip models can be established.

For medium reservoir modules, its bottom PMMA sheet should be thick enough to fabricate the 90° elbow medium access ports inside it (Figure 2b). Four PMMA sheets were then glued to four sides of the bottom sheet and sealed with PDMS after curing. The magnet was glued on one side and interconnected with the medium access port. For the design with only one medium access port, multiple medium access ports could be integrated on a single medium reservoir module for high throughput tissue culturing, as shown in Figure 2c.

### 2.4. Assembly of RSUIM with the Generic Organ-on-a-Chip Module

**Figure 3:**
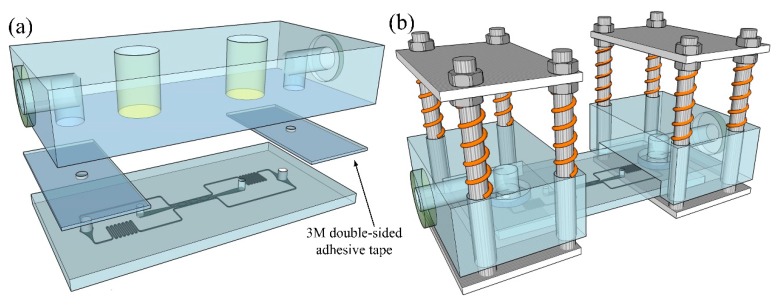
Schematic of the assembled RSUIM with the generic organ-on-a-chip module. (**a**) Pasting-based RSUIM assembly with 3M double-sided transparent adhesive tape. (**b**) Clamping-based RSUIM assembly by the clamping force from springs.

For the pasting-based RSUIM assembly, 3M acrylic double-sided transparent adhesive tape (3M, Shanghai, China), with a thickness of 1 mm was utilized as the sealing gasket, as shown in Figure 3a. Holes with the diameter of 2 mm were punched at each adhesive tape, whose sizes were slightly larger than those of the medium perfusion ports on the organ-on-a-chip module, but much smaller than those of the medium access ports on the RSUIM for easy assembly. It should be noted that due to the inherent low adhesive capability between adhesive tape and the PDMS-based organ chip, the surface of the organ chip should be pre-treated with special treatment agent for silicon rubber (XH-211, Xinhui company, Wuhan, China), which enabled tight adhesion after the treatment.

For the clamping-based RSUIM assembly, tight adhesion would be much easier, as shown in Figure 3b. By pressing the lower PMMA sheet downwards, the organ-on-a-chip module was inserted into the groove. The medium perfusion port on chip module was then aligned and fixed under the O-ring by releasing the lower PMMA sheet. The clamping force was imposed by four springs, which could be simply adjusted by moving the upper PMMA sheet up and down on the screws.

### 2.5. Holding Strength Characterization of Interconnected RSUIM

The holding strength of interconnected RSUIM through a magnet was characterized by using a tensile test machine (SH-50, SUNDOO, Wenzhou, China). Briefly, the interconnected devices were fixed in the clamps of the tensile test machine and withstood the increased separation force. The maximum holding force was defined as the force required to separate these two RSUIMs completely. For comparison, the holding strengths of both syringe needle PDMS and magnet-gasket-magnet modules were measured in the same way.

### 2.6. Fluid Leakage Testing of Assembled Modular Devices

Using either the circle profile O-ring or 3M double-sided transparent adhesive tape as the sealing gasket, as well as the interconnected RSUIMs through the magnets, the maximum fluid pressure was measured by using a syringe pump, which was performed by pumping water through tubing and into the interconnected modules with an increased flow rate until the fluid leakage was first observed at their interfaces.

### 2.7. Cell Culture and ECM Preparation

The GFP-expressing human umbilical vein endothelial cells (GFP-HUVECs) were purchased from Angio-proteomie (Boston, MA, USA) and cultured in EGM-2 (Lonza, Basel, Switzerland), which were used before passage 6. Normal human lung fibroblasts (NHLFs) were obtained from Lonza (Basel, Switzerland) and cultured in the fibroblast medium (FM, Sciencell, Carlsbad, CA, USA) to passage 6 before use. All cell types were cultured in a 37 °C/5% CO_2_ incubator in 100% humidified air environment.

After harvesting cells from a flask, GFP-HUVECs, at the concentration of 10 × 10^6^ cells per mL, and NHLFs at the concentration of 6 × 10^6^ cells per mL, were suspended in fibrinogen solution (Sigma-Aldrich, St. Louis, MI, USA) with a concentration of 10 mg/mL. After mixing with 50 U/mL thrombin (Sigma-Aldrich, St. Louis, MI, USA) for a final concentration of 3 U/mL, the mixed ECM was quickly injected into the tissue chamber through gel loading inlet ports by using a micropipettor.

### 2.8. Sterilization and Medium Perfusion into Assembled Modular Devices

For sterilization, each module was autoclaved at 121 °C for 30 min before being used for cell/tissue culture experiments. For medium perfusion after gel loading, 1 mL of cell culture medium was injected into the assembled modular devices from medium access ports on the RSUIM by using a micropipettor until no air bubbles appeared inside the microfluidic channels. To interconnect two RSUIMs or the RSUIM with the medium reservoir modules, a small drop of cell culture medium was dripped onto the magnetic ports, and these two droplets were carefully merged together to avoid bubbles.

### 2.9. Finite Element Simulation

Finite element simulations for both interstitial flow through ECM embedded inside the tissue chamber and flow profile inside the vessel lumen were performed using COMSOL Multiphysics 4.3 (Comsol Inc., Burlington, MA, USA). For the simulation of the generic vascularized organ-on-a-chip module, the Brinkman’s equation was employed for momentum transportation through a porous fibrin gel with a low permeability of 1.5 × 10^−13^ m^2^ and a porosity of 0.99, and the hydrostatic pressure drop between the medium inlet and outlet was set to be 10 mm H_2_O. For the simulation of flow profile inside the vessel lumen, the Navier–Stokes equation was utilized to model the real microvascular network formed inside tissue chambers. Briefly, the fluorescent image was first binarized and then performed various image processing, such as eroding and dilating to fill holes inside vessels and remove noises by using ImageJ (Version 1.51j8, National Institutes of Health, Bethesda, MD, USA). Its contour was then extracted by using an edge detection algorithm and converted into vectorized images by using Img2CAD software (Version 7.3, Cologne, Germany), which could be imported into COMSOL. The blood viscosity of 3 cP was utilized in the simulation, and the hydrostatic pressure drop between the microvessel inlet and outlet was set to be 5 mm H_2_O.

### 2.10. Imaging and Analysis

Fluorescent images were taken using a Leica inverted fluorescence microscope with a CCD camera (Nikon, Tokyo, Japan). For vessel quantitative analysis, both the vessel percentage area and average vessel length were characterized using AngioTool software (Version 0.6a, National Cancer Institute, Bethesda, MD, USA).

## 3. Results

### 3.1. Fabrication and Assembly of RSUIM Devices

**Figure 4:**
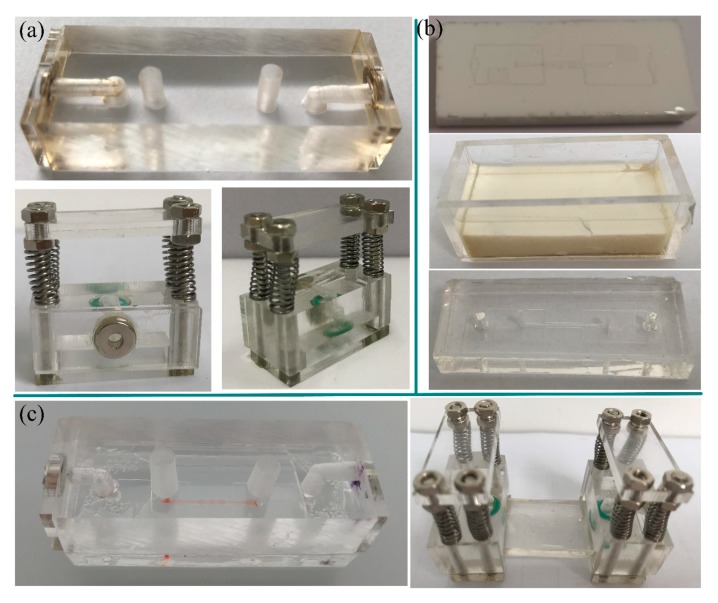
Fabrication and assembly of RSUIM devices. (**a**) Prototype of fabricated pasting-based RSUIM (**top**) and clamping-based RSUIM (**bottom**). (**b**) Prototype of the generic vascularized organ-on-a-chip module (**bottom**) through a customized polyurethane master mold (**top**) inside a customized poly(methyl methacrylate) (PMMA) frame (**middle**). (**c**) Protype of assembled pasting-based (**left**) and clamping-based (**right**) RSUIMs with the fabricated generic vascularized organ-on-a-chip module.

Since it would be costly and cumbersome to fabricate the magnetic interconnect by embedding and/or fixing magnets into microfluidic chips, the developed RSUIM provides a low-cost and convenient solution to achieve a robust and flexible interconnection between different modular chips. Thus, the basic requirement for these RSUIMs should be recyclable, easy to fabricate, and most importantly, consistent and reliable after reusing several times. Due to the transparent property and mature processing technique using laser cutting, PMMA is the optimal material to fabricate the RSUIM. Figure 4a shows the prototype of pasting-based RSUIM and clamping-based RSUIM, respectively. Furthermore, since the dimensions of the organ-on-a-chip module will directly determine the parameters of the RSUIM, it is necessary to realize the standardization and universalization of the chip module. Here, a customized polyurethane master mold of the microfluidic chip was fabricated using 2-part polyurethane liquid plastic (Smooth Cast 310, Smooth-On Inc., Macungie, PA, USA) [17], as shown in Figure 4b. The whole size of the organ-on-a-chip module was determined by a customized PMMA frame, and the microfluidic chip was casted by pouring PDMS into it. Figure 4c shows the assembled pasting-based RSUIM and clamping-based RSUIM with the fabricated generic vascularized organ-on-a-chip module, respectively. The polarity of the magnetic interconnect in each assembled RSUIM was kept to be the opposite, which was convenient for the future interconnection of different assembled RSUIMs.

### 3.2. Characterization Results

**Figure 5:**
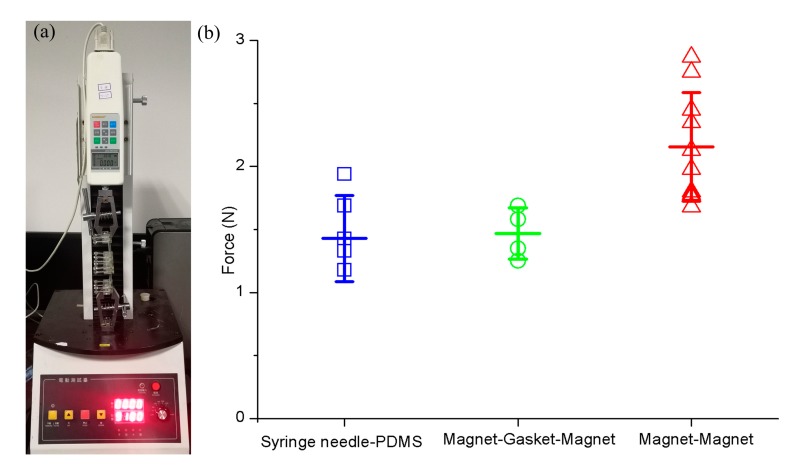
Characterization on attraction strength among different connection modes. (**a**) Tensile test machine. (**b**) Characterization results.

Compared with the conventional connection mode, such as syringe needle-PDMS or magnet-gasket-magnet, the attraction strength for magnet-magnet in our assembled RSUIM device was relatively higher, and the average force required to separate two magnet-magnet modules was 2.16 ± 0.43 N, which was characterized by using the tensile test machine (Figure 5). Furthermore, a fluid leakage testing was performed to ensure no leakage occurred when perfusing the culture medium through the magnetic interconnectors, and the maximum hydraulic pressure withstood these interconnects before leakage and could reach up to 2.17 × 10^4^ Pa when connecting plastic tubing at the length of 4 cm and an inner diameter of 100 µm through the syringe pump at the volumetric flow rate of 90 μL/min. Moreover, at this hydraulic pressure, due to the strong adhesion of 3M double-sided tape and tight clamping force imposed by the springs, there was no leakage observed at the interface between the organ chip module and RSUIM in the pasting-based and clamping-based designs, respectively.

### 3.3. Vascularization inside the Assembled Modular Device

**Figure 6:**
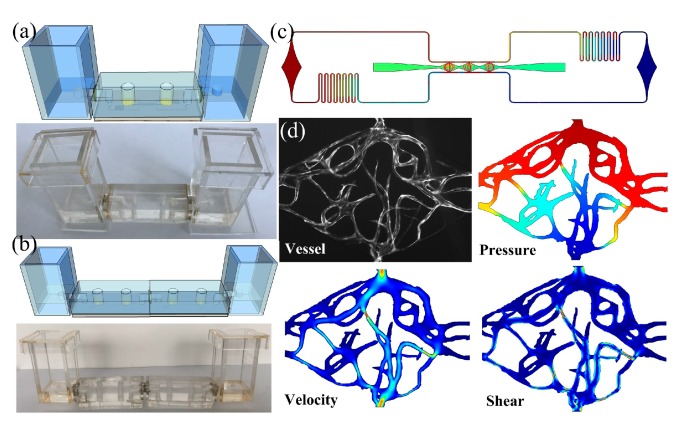
Vascularization inside the assembled RSUIM device. (**a**) Schematic and prototype of the assembled pasting-based RSUIM device with two medium reservoir modules. (**b**) Schematic and prototype of two assembled modular devices with the serial connection mode. (**c**) Simulation results of the uniform interstitial flow profile inside the tissue chambers of the generic vascularized organ-on-a-chip with coupling design. (**d**) Formation of fluorescent microvascular network inside the tissue chamber and its corresponding simulation results on the pressure distribution, velocity profile, and shear stress inside the vessel lumen, respectively.

In order to test the feasibility of this assembled modular device, especially in organ-on-a-chip applications, we performed the vascularization within the fabricated generic organ-on-a-chip modules by the combination of both the medium reservoir module and the pasting-based RSUIM, as shown in Figure 6a. Furthermore, Figure 6b shows the schematic and prototype of two assembled modular devices with the serial connection mode. After injecting the cell-seeded ECM into tissue chambers from the gel loading inlet port, the hydrostatic pressure drop of 10 mm H_2_O was established between the inlet and outlet medium reservoir modules. Figure 6c shows the simulation results of uniform interstitial flow inside tissue chambers within the optimal range (0.1–11 μm/s) to continuously induce vasculogenesis. Figure 6d shows the formed microvascular network inside one tissue chamber and its simulation results on the flow profile inside vessel lumen. The hydrostatic pressure drop between its top inlet and bottom outlet was set to be 5 mm H_2_O. The average flow velocity and shear stress was around 300 μm/s and 2.5 dyn/cm^2^ by using the viscosity coefficient of blood, which was in accordance with the microphysiological environment of the human capillary network in vivo.

### 3.4. Highly Reproducible Vascularization in a High Throughput Platform

**Figure 7:**
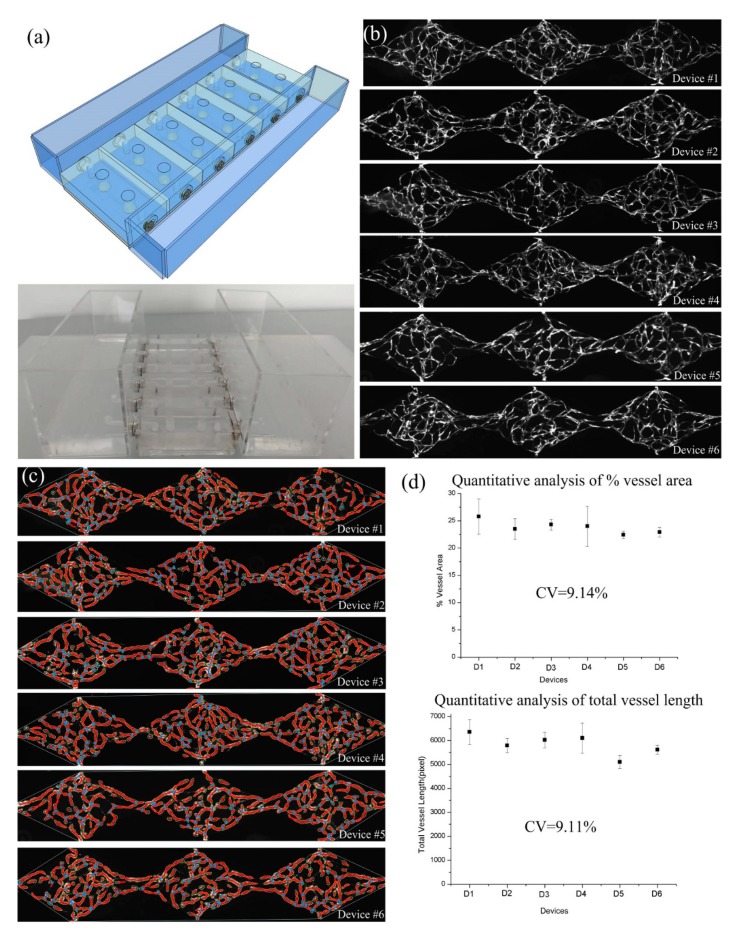
Highly reproducible vascularization inside the high throughput platform with six parallel pasting-based assembled modular devices. (**a**) Schematic and prototype of the assembled high throughput platform. (**b**) Formation of the fluorescent microvascular network inside six vascularized organ-on-a-chip modules. (**c**) Analysis results on the percent vessel coverage and total vessel length using AngioTool software. (**d**) Quantification results on the percent vessel coverage and total vessel length with high consistency.

Due to the flexibility of the modular design, it was simple to integrate multiple assembled modular devices in a high throughput manner. Figure 7a shows the parallel culturing within six parallel pasting-based assembled modular devices connected to two medium reservoir modules with multiple medium access ports. Successfully loaded devices could develop robust and uniform vascular networks within seven days, as shown in Figure 7b. Furthermore, the vessel area percentage and total vessel length inside each tissue chamber were analyzed to assess the reproducibility of the vascular formation in each successfully loaded device on the same platform (Figure 7c). Quantification results demonstrated that the percent vessel coverage and total vessel length between the tissue chambers (n = 18 tissue chambers) on a single platform (n = 6 devices) were highly consistent, with a coefficient of variation (CV) equal to 9.14% and 9.11%, respectively (Figure 7d).

### 3.5. The Vascularized Tumor Model and its Application in Anti-cancer Drug Screening

**Figure 8:**
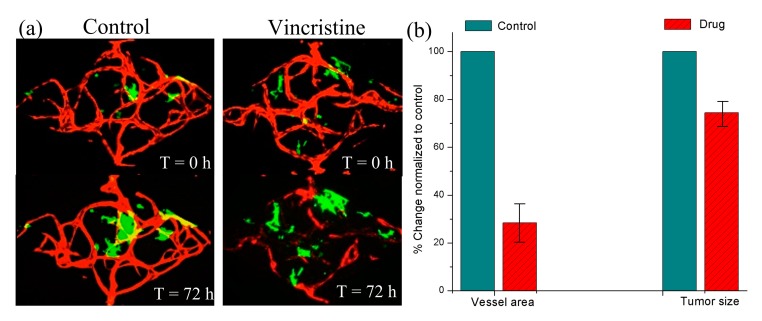
Vascularized tumor model formation inside the assembled pasting-based RSUIM device by co-culturing tumor spheroids with the microvascular network. (**a**) A contrast experiment by adding the anti-cancer drug into the developed vascularized tumor model. (**b**) Quantitative analysis on the vessel area and tumor size before and after adding the drug.

In addition to vascularization, we also realized the vascularized tumor model through the assembled pasting-based RSUIM device by co-culturing tumor spheroids with the microvascular network, as shown in Figure 8a. In the control group, due to the continuous cell culture medium perfusion, both the microvascular network and tumor spheroids grew gradually. Specifically, the microvascular network became mature and lumenized, while the size of the tumor spheroids increased after 72 h. However, after adding vincristine as the experimental group, which is known as an anti-cancer chemotherapy drug to destroy tumor blood vessels, the change of microvascular area and tumor size decreased to 26.4 ± 8.65% and 68.9 ± 5.09% normalized to the control group (n = 4), as shown in Figure 8b. The preliminary experimental results were in accordance with its clinical testing results.

## 4. Discussion

By integrating multiple organs/tissues onto a single platform, multi organ-on-a-chip with 3D microstructure and specific microphysiological functions can better provide a more accurate model of the human body, which can be widely used in drug development, disease modeling, and toxicological screening, as well as personalized medicine. The modular-based multi-organ integration enables more stable and flexible configuration due to its temporal flexibility, decoupled modules, and redundancy capabilities, which can help to solve the common problems existing in the multi organ-on-a-chip applications, such as scaling criteria, allometric growth, and universal culture medium. In addition to these advantages, our developed RSUIM can be used multiple times in a plug-and-play manner, which can provide a low-cost and convenient solution to achieve a robust and flexible interconnection of different modular chips.

Besides the fabrication with PMMA, the RSUIM can also be designed and fabricated through 3D printing for a more complex structure. Similarly, in addition to PDMS, the generic organ-on-a-chip modules can also be made from thermoplastic, such as cyclic olefin copolymer (COC), PMMA, or polycarbonate, with the processing technologies of either hot embossing or injection molding, which is the optimal selection in drug screening applications due to its prevention of small molecule absorption. Here, for the generic vascularized organ-on-a-chip module with the coupling design, since there was only one inlet and outlet, two magnetic interconnects were fabricated on RSUIM. Moreover, RSUIM with multiple magnetic interconnects can be extended to other organ-on-a-chip modules with multiple inlets and outlets based on different applications. Besides the basic configuration of the organ-on-a-chip module and medium reservoir module, other functional modules, such as the bubble trap module, online-sensor module, or fluidic control module, can also be flexibly plugged into the system when needed. In our preliminary experiment, only the microvascular network or vascularized microtumor model was constructed for the drug screening application. In future work, multiple different organs/microtissues can be interconnected in series through the microvascular network to model more complex biological phenomena, such as multi-organ interactions, drug pharmacodynamics, and pharmacokinetics (PD-PK) or systemic toxicity side effects.

## 5. Conclusions

In this paper, we have presented both pasting-based and clamping-based RSUIM for generic organ-on-a-chip applications. The configurable plug-and-play concept-based modular design is simple to use, easy to manufacture, reversible, and reliable. Most importantly, this modular design can be utilized multiple times, which is improves commercialization with its low cost. Vascularized microtissues grown inside the assembled RSUIM with the generic organ-on-a-chip module are highly reproducible, and the formed microvascular network is fully functional. Furthermore, quantitative analysis results on its reproducibility and validation in anti-cancer drug screening were further performed to test the effectiveness of this RSUIM. This module design will greatly facilitate the integration of the versatile organ-on-a-chip module into a multi organ-on-a-chip or high throughput platform, which has the potential to become a standard and critical world-to-chip interface, as well as the interorgan connectors, for a broad range of organ-on-chip applications.
